# The Effect of Neighborhood Cohesion Across the Lifespan on Cognitive Health

**DOI:** 10.1093/geroni/igaa057.1284

**Published:** 2020-12-16

**Authors:** Elizabeth Munoz, Yee To Ng, Sae Hwang Han

**Affiliations:** The University of Texas at Austin, Austin, United States

## Abstract

Neighborhood environments may serve as protective factors against cognitive impairment and decline. Recent evidence shows that neighborhood cohesion is associated with better cognitive health in adulthood. We extend the current literature by evaluating how neighborhood cohesion across the lifespan may influence cognitive function in adulthood. We used data from the Health and Retirement Study (HRS) and the HRS Life History Survey. Participants who were 50-89 years old at baseline, completed up to 10 longitudinal waves, and were not cognitively impaired in the last wave were included in the analyses (N=2,057). Early-life neighborhood cohesion was assessed with participants’ retrospective ratings of sense of belonging in their local areas at age 10, when they first had a full-time job, and at age 40. Participants’ assessment of neighborhood cohesion assessed at the final wave was treated as the current indicator of cohesion. Cognitive function was assessed with the full HRS cognitive battery. Preliminary findings from hierarchical mixed models showed an overall decline in cognitive function across time. Current and age 10 neighborhood cohesion were independently associated with better baseline cognitive function, but did not predict cognitive change. Interaction terms involving cohesion at age 10 and current neighborhood cohesion showed that current cohesion buffered the negative effect of low cohesion at age 10. No significant associations were observed for cohesion during the full-time job and age 40 periods. Although effect sizes were small, these results indicate that neighborhood cohesion in older adulthood may offset the detrimental effects of negative childhood environments on cognitive health.

